# Metal-deficient aggregates and diminished copper found in cells expressing SOD1 mutations that cause ALS

**DOI:** 10.3389/fnagi.2014.00110

**Published:** 2014-06-16

**Authors:** Megan W. Bourassa, Hilda H. Brown, David R. Borchelt, Stefan Vogt, Lisa M. Miller

**Affiliations:** ^1^Department of Chemistry, Stony Brook UniversityStony Brook, NY, USA; ^2^Department of Neuroscience, Center for Translational Research in Neurodegenerative Disease, Santa Fe Health Care Alzheimer’s Disease Research Center, McKnight Brain Institute, University of FloridaGainesville, FL, USA; ^3^Advanced Photon Source, Argonne National LaboratoryArgonne, IL, USA; ^4^Photon Sciences Directorate, Brookhaven National LaboratoryUpton, NY, USA

**Keywords:** amyotrophic lateral sclerosis, superoxide dismutase, X-ray fluorescence microscopy, synchrotron

## Abstract

Disruptions in metal ion homeostasis have been described in association with amyotrophic lateral sclerosis (ALS) for a number of years but the precise mechanism of involvement is poorly understood. Metal ions are especially important to familial ALS cases caused by mutations in the metalloenzyme copper-zinc superoxide dismutase (SOD1). To investigate the role of metals in aggregation of mutant SOD1, we have examined the localization of metal ions in a cell culture model of overexpression. Chinese hamster ovary cells (CHO-K1) were transfected to overexpress SOD1 fused to yellow fluorescent protein (YFP) to readily identify the transfected cells and the intracellular aggregates that develop in the cells expressing mutant or wild-type (WT) SOD1. The concentration and distribution of iron, copper, and zinc were determined for four SOD1 mutants (A4V, G37R, H80R, and D125H) as well as a WT SOD1 using X-ray fluorescence microscopy (XFM). Results demonstrated that the SOD1 aggregates were metal-deficient within the cells, which is consistent with recent *in vitro* studies. In addition, all SOD1 mutants showed significantly decreased copper content compared to the WT SOD1 cells, regardless of the mutant’s ability to bind copper. These results suggest that SOD1 overexpression creates an unmet demand on the cell for copper. This is particularly true for the SOD1 mutants where copper delivery may also be impaired. Hence, the SOD1 mutants are less stable than WT SOD1 and if copper is limited, aggregate formation of the metal-deficient, mutant SOD1 protein occurs.

## Introduction

Amyotrophic lateral sclerosis (ALS) is a neurodegenerative disease that affects more than 30,000 people annually in the US, making it the most common motor neuron disease (Bruijn et al., 2004). ALS begins with muscle weakness that develops into progressive paralysis and eventually leads to death, which generally occurs within 2–5 years of the disease diagnosis. There is currently no known cure for the disease and the limited treatments available do not significantly alter the course of the disease (Miller et al., 2007). Approximately 90% of all ALS cases are sporadic in nature with no known cause. The remaining 10% have a genetic link and are known as familial ALS cases. One such form of familial ALS, which accounts for 2.5% of all ALS cases, is caused by mutations in the gene that codes for the antioxidant protein copper-zinc superoxide dismutase (SOD1).

SOD1 is a metalloenzyme that binds one copper ion and one zinc ion per monomer for the protein’s activity and stability, respectively (Ellerby et al., 1996). There are over 160 known mutations in SOD1 that can cause ALS (Lill et al., 2011; Abel et al., 2013). Despite extensive research, it is currently not understood how such a large distribution of mutations and their properties can all result in the same disease. These mutations are distributed throughout the protein and result in SOD1 mutations with dramatically varied biochemical properties, such as metal binding affinity and antioxidant activity levels. Thus, mutations are classified into two distinct categories: metal binding region (MBR) mutations, which directly affect the protein’s ability to bind metal causing diminished activity levels, and wild type-like (WTL) mutations, which maintain metal binding ability and have activity levels similar to that of the wild-type (WT) protein (Hayward et al., 2002; Rodriguez et al., 2002). In this study, four SOD1 mutations were evaluated, including WTL mutations (A4V and G37R) and MBR mutations (H80R and D125H). The A4V mutation occurs at the SOD1 dimer interface and has very low stability (Deng et al., 1993; Hayward et al., 2002), whereas the G37R mutation is in an electrostatic loop and has much higher stability (Borchelt et al., 1994). The H80R mutation is found in the zinc-binding region, hence zinc binding is diminished (Seetharaman et al., 2010). Since the ability of SOD1 to bind copper is thought to be dependent on its ability to bind zinc first, this mutation is also deficient in copper. The D125H mutation occurs in a charged loop that helps coordinate the metal binding site (Hayward et al., 2002). While it does not directly affect metal-binding, this mutation severely diminishes metal binding affinity and results in a protein with low stability.

Metals have been implicated in a number of neurodegenerative diseases including ALS (Bush, 2003; Maynard et al., 2005; Vonk and Klomp, 2008). When improperly regulated, metal ions such as copper and zinc can be highly toxic to the cells. Copper is especially dangerous due to its catalytic activity and redox potential, resulting in the production of damaging reactive oxygen species (ROS), which lead to the propagation of oxidative stress found in patients with ALS (Bruijn et al., 2004; Lutsenko et al., 2010). Alterations in zinc concentrations can also cause problems such as glutamate excitotoxicity, in which excess zinc over stimulates neurons resulting in cell death (Smith and Lee, 2007). Interestingly, Riluzole, the only approved drug for the treatment of ALS, reduces excitotoxicity by inhibiting glutamate release (Doble, 1996). Thus, proper metal homeostasis is critical to maintaining a healthy cellular environment and delaying disease progression.

A common pathological feature of SOD1-ALS is the formation of misfolded SOD1 aggregates in the spinal cord motor neurons that form concomitantly with the onset of paralysis. Protein aggregates are found in several other neurological diseases, such as the amyloid-β plaques found in Alzheimer’s disease (AD) and Lewy bodies composed of α-synuclein in Parkinson’s disease (PD; Selkoe, 2003, 2004; Soto, 2003, 2013). The role of these aggregates in disease has been the subject of great debate for a number of years. Originally, aggregates were thought to be the source of toxicity in these diseases. However, it has also been suggested that the aggregates are a cellular response to sequester toxic soluble proteins, avoiding further damage from the misfolded and aberrant proteins (Lee et al., 2002).

The mechanisms of aggregation are not well understood, but metal ions have been implicated. For example, it has been observed that metals can aid in the precipitation and aggregation of amyloid-β (Huang et al., 1997) and α-synuclein (Bertoncini et al., 2005). It was recently shown that the SOD1 aggregates found in a mouse model of SOD1-ALS are composed of unmodified and nascent SOD1 (Shaw et al., 2008). Nascent and unmetallated SOD1 mutants are relatively unstable compared to nascent WT SOD1, suggesting that mutants are more prone to aggregation than WT SOD1 (Rodriguez et al., 2002, 2005).

In this study, we used X-ray fluorescence microscopy (XFM) to image the metal ion distribution and the metallation state of the SOD1 aggregates in Chinese hamster ovary (CHO-K1) cells overexpressing WT SOD1 or mutant SOD1 (A4V, G37R, H80R and D125H). XFM is a spectroscopic technique that can be used to determine the concentration and distribution of metal ions in biological cells and tissues. To visualize the transfected cells and the aggregates, SOD1 was fused in-frame with yellow fluorescent protein (YFP) (Prudencio et al., 2009; Qualls et al., 2013a,b). Results showed that the SOD1 aggregates were metal-deficient within the cells, which is similar to the detergent-insoluble aggregates isolated from a transgenic mouse model of SOD1-ALS (Lelie et al., 2011). In addition, we found that cells with mutant SOD1 contained a lower level of copper compared to cells expressing WT SOD1, regardless of whether the SOD1 mutant was capable of binding copper. These results were surprising and suggest that copper transport is affected regardless of the SOD1 mutation.

## Materials and methods

Chinese hamster ovary cells (CHO-K1) (ATCC, Manassas, VA) were maintained in F12-K media with 10% fetal bovine serum, 1% penicillin/streptomycin, and 1% amphotericin- β. The cells were stored in an incubator at 37°C and 5% CO_2_. Cells were seeded onto 2.5 × 2.5 mm silicon nitride windows (Silson, UK), an IR and X-ray transparent material, with a 500 nm membrane thickness. After 20 h, when the cells were approximately 80% confluent, they were transfected with plasmid cDNA (Karch et al., 2009) using lipoD293 transfection reagent (SignaGen, Rockville, MD) according to the protocol provided by SignaGen. CHO-K1 cells were transfected to express a WT or mutant (A4V, G37R, H80R, or D125H) form of SOD1 fused to a YFP tag to visualize the transfected cells and aggregates without staining. The transfections were stopped between 19–26 h, which was when YFP fluorescence was visible in most cells and punctate aggregates could be seen (Prudencio et al., 2009). The time to completion was varied so that the aggregate levels would be equivalent for each mutation used. Cells were fixed by rinsing the silicon nitride windows with PBS and then dipping in cold (~80°C) methanol three times for 1 s each time. The windows were allowed to dry at room temperature and were stored in a desiccator until imaged. Each transfection was carried out twice, and cells from both transfections were imaged.

FTIR microspectroscopy was used to determine the protein density of the aggregates. Spectra were acquired using a Thermo Nicolet Continuum IR microscope coupled to beamline U2B at the National Synchrotron Light Source (NSLS) at Brookhaven National Laboratory (Upton, NY). A 32 × IR Schwarzchild objective produced a 7 × 7 μm beam. A 4 cm^−1^ spectral resolution and 128 scans per spectrum were used over the mid-infrared region (4000–800 cm^−1^). To normalize the additional protein density found in the protein aggregates, 40–45 spectra were collected both on the aggregates and off the aggregates of transfected cells. For each spectrum, the protein content was determined by integrating the Amide I band from 1610–1700 cm^−1^ with a linear baseline from 1480 to 1800 cm^−1^. The relative protein intensity was determined by calculating the intensity ratio on the aggregates versus off the aggregates for each cell.

The cells were then imaged with XFM at beamline 2-ID-E at the Advanced Photon Source at Argonne National Laboratory (Argonne, IL) to determine the distribution and concentration of metal ions. The incident beam was tuned to 10 keV using a Si(111) monochromator and the X-rays were focused to a 0.5 × 0.5 μm spot size using 20 cm zone plates (Xradia, Pleasanton, CA). The specimen was positioned at a 45° angle with respect to the incident beam and maintained in a helium-purged box. The X-ray fluorescence was detected with a four-element silicon drift detector oriented at 90° with respect to the incident beam and a differential phase contrast detector was used for the transmitted X-ray beam. Approximately 15 cells were imaged per mutation with a 0.5-s dwell time per point. A full energy dispersive spectrum was collected at each pixel. Metal concentrations were determined based on NIST thin film standards 1832 and 1833.

To analyze the data, three regions of interest (ROI) were generated using the beamline’s MAPS program: (1) the nucleus; (2) the cytoplasm; and (3) the aggregates (if present). The cytoplasm ROI was separated from the nucleus to provide a more accurate measure of metal concentrations in the cytoplasm where the SOD1 is located. The aggregates were easily located using the phase contrast images collected with the XFM data. In addition, the aggregates were small (1–3 μm) so the 500 nm beam size allowed for several pixels (approximately 10) from each aggregate to be analyzed.

After generating ROIs, the data points within each ROI were averaged and the aggregate values were normalized to the protein density as determined by FTIR. Using the average values, a ratio of the aggregate/cytoplasm was generated for copper and zinc to demonstrate any change in metal content within the aggregates. Statistics were performed on mean values from the ROIs of each cell type with SPSS software (version 14.0) using the Kruskal-Wallis non-parametric test. Significant groups were then tested with Mann-Whitney *U* *post-hoc* test. Significance was determined by *p* < 0.05.

## Results

**Figure 1:**
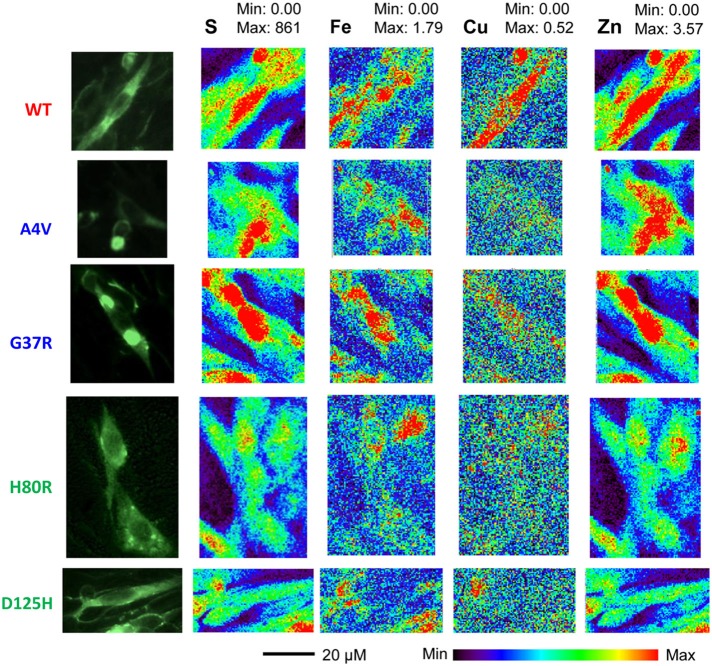
**Epifluorescence images (first column) from SOD1-YFP CHO-K1 cells with WT, A4V, G37R, H80R or D125H SOD1**. XFM maps for sulfur (second column), iron (third column), copper (fourth column), and zinc (fifth column). The XFM images show the relatively large amounts of copper and zinc found in the WT cells compared to the mutant SOD1 cells. Minimum and maximum concentrations are in mM.

**Figure 2:**
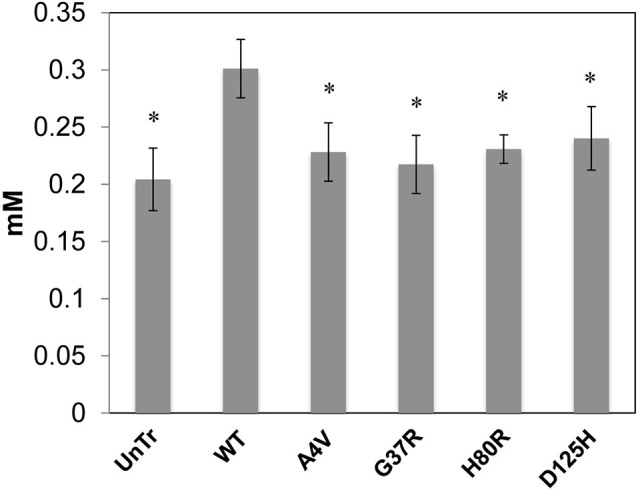
**Bar graph of intracellular copper levels (excluding the nucleus) for SOD1-YFP cells**. Concentrations are in mM. The WT cells contained the highest level of copper in comparison to the cells overexpressing mutations in SOD1 and untransfected cells. All cells overexpressing mutant SOD1 had significantly higher copper content than the untransfected cells. * indicates significantly less than WT cells with *p* < 0.001.

Representative XFM and visible light images of the cells from each group (WT, A4V, G37R, H80R, and D125H) are shown in Figure 1. As can be seen, the iron concentration was higher in the cytosol, while the zinc content was highest in the nucleus for all cell types. The only statistically significant difference between the cell types was found in the copper content in the cytoplasm. Specifically, the WT cells contained approximately 24% more copper than all of the SOD1 mutants (A4V, G37R, H80R and D125H) as well as the untransfected cells (Figure 2). The mutant SOD1 cells also contained a smaller but significant (*p* > 0.05) increase in copper compared to the untransfected cells. Interestingly, there were no statistical differences between the mutant SOD1 cells, regardless of metal-binding ability. Also, no trends were observed in the zinc, iron, phosphorus, sulfur, potassium or calcium concentrations between the untransfected and transfected cells in the cytoplasm or the nucleus.

Based on the FTIRM data, the aggregates showed a two-fold increase in protein density compared to the surrounding cytoplasm. Thus, the XFM data for the protein aggregates were normalized by this factor to account for the greater amount of material within the aggregates. Table 1 shows the ratio of aggregate to cytoplasm concentrations for copper and zinc from cells containing mutant SOD1-YFP (A4V, G37R, and H80R) aggregates. Results showed that all of the aggregates of mutant SOD1-YFP contained lower copper and zinc concentrations compared to the surrounding area, suggesting that the aggregates are metal-deficient. This trend was seen in both the WTL (A4V and G37R) and MBR (H80R and D125H) mutations.

**Table 1:** **The ratio of aggregate/cytoplasm concentration for copper and zinc in the SOD1 mutant cells**.

**Mutation**	**Type**	**Copper**	**Zinc**
A4V	WTL	0.53 ± 0.13	0.34 ± 0.21
G37R	WTL	0.45 ± 0.081	0.59 ± 0.27
H80R	MBR	0.41 ± 0.041	0.31 ± 0.14

## Discussion

### SOD1 aggregates in cells are metal-deficient

The formation of SOD1 aggregates in the spinal cord is the primary pathology found in ALS patients with SOD1 mutations, but little is known about the aggregation process. The precise mechanism of aggregate formation is needed in order to gain an understanding of how aggregates impact disease progression. Cells transfected with mutant SOD1 develop intracellular aggregates, similar to those found in ALS patients. The results presented here show that the SOD1 aggregates in the transfected cells are metal-deficient. This was true regardless of the mutant’s ability to bind copper (WTL and MBR mutants). These data also agree with a previous study that showed insoluble or aggregated SOD1 extracted from ALS mouse spinal cords contained no metal (Lelie et al., 2011). These data suggest that either the protein aggregated prior to being metallated or that the process of aggregation resulted in a loss of metal.

The aggregation of nascent SOD1 prior to metallation is also supported by the very high stability of most metallated SOD1 mutations, making them less likely to aggregate once they are metallated (Hayward et al., 2002; Rodriguez et al., 2002). In contrast, apo-SOD1 is significantly less stable and contains large, intrinsically disordered regions that make the protein much more prone to aggregation (Rodriguez et al., 2005; Shaw and Valentine, 2007; Lelie et al., 2011).

In contrast to SOD1-ALS, protein aggregates found in other neurodegenerative diseases, including amyloid-β plaques in AD and Lewy bodies in PD, have been shown to have a high metal content (iron, copper or zinc) (Selkoe, 2001; Lotharius and Brundin, 2002; Leskovjan et al., 2009). For example, AD plaques, which consist primarily of aggregated amyloid-β protein and form in the brain of human patients, were found to be highly enriched in metal (calcium, iron, copper and zinc) compared to the surrounding brain tissue (Lovell et al., 1998; Miller et al., 2006). However, in a mouse model of AD, it was found that the plaques did not contain elevated metal ions (Leskovjan et al., 2009). This has led to the hypothesis that AD plaques may accumulate metal over time. For ALS, the studies presented here were limited to a cell culture model of the disease. In the future, further studies from the mouse model and/or human tissue will be needed to determine if the mechanism of protein aggregation and the role of metals is similar to other neurological diseases or unique to ALS.

### Copper concentration is lower in cells overexpressing SOD1 mutations

The WT cells overexpressing SOD1 contained significantly more copper than the untransfected cells. This increase in copper content demonstrates that there is presumably enough copper available to the cells to metallate the expressed WT SOD1.

To investigate the effects of mutant SOD1 on metal homeostasis, this study utilized two different types of SOD1 mutations including WTL mutations (A4V and G37R), which are known to bind copper and zinc, and MBR mutations (H80R and D125H), which are unable to bind copper or zinc (Rodriguez et al., 2002, 2005; Valentine et al., 2005). Results showed that all mutations contained approximately 24% less copper than the WT cells. In addition, all mutants contained similar copper concentrations regardless of metal-binding ability.

For WT SOD1, previous studies showed a reduced incorporation of copper when over-expressed in cell culture (Prudencio et al., 2012). Here we show that the copper content in the WT SOD1 cells is significantly higher than the untransfected cells, but also suggests that that there is still an insufficient supply of copper to metallate the overexpressed level of SOD1. For the mutant SOD1 in this cell culture model, a substantial fraction of the protein adopts a non-native conformation that remains soluble and freely mobile (Prudencio and Borchelt, 2011) and decreased copper binding has also been observed (Ayers et al., 2014). Thus, it is likely that the lower copper content in all the cells transfected with mutant SOD1 is due to a combination of insufficient copper delivery to the cells and inefficient copper binding to the active site.

Delivery of copper to the cells is a highly regulated process. Due to its potential redox activity, copper can be highly toxic when not properly controlled. As a result, copper transporters and chaperone proteins tightly regulate the concentration of copper in the cell. Maintaining a strict copper balance is critical and a disruption in homeostasis is implicated in several diseases, including ALS (Gaggelli et al., 2006). For SOD1, copper is transported by the copper chaperone protein (CCS). CCS contains an SOD1-binding domain that has approximately a 50% amino acid sequence homology to SOD1, which is important for the recognition and binding of SOD1 (Schmidt et al., 1999). All four mutations examined in this study have residues that reside in the homologous binding domain of CCS, which may prevent the efficient binding of CCS to SOD1, possibly altering the transport of copper. While CCS levels were not measured in this study, future studies will address the question of copper transport directly.

Taken together, our results suggest a relationship between the metal-deficient aggregates and the decreased copper found in the cells overexpressing mutant SOD1. We suggest that SOD1 overexpression creates an added demand on the cell for Cu, which is not met for the WT or mutant SOD1. In the mutant SOD1, impaired copper transport to the protein may even further reduce the copper content in the cell. Since the mutant proteins are less stable than WT SOD1, and copper is less available, aggregate formation of the metal-deficient mutant SOD1 protein occurs.

One limitation of this work is that it utilized a cell culture model, which does not always correlate with the disease *in vivo*. That said, the data presented here agree with our previous studies on insoluble aggregates extracted from mouse spinal cords, which were found to be metal-deficient. In the future, it will be important to examine the metal content of these aggregates directly within the spinal cord tissue of mouse models of SOD1-ALS. Levels of the CCS and its ability to bring copper into SOD1 also need to be assessed. Lastly, measuring aggregates in human tissue would be the definitive experiment, but the SOD1 mutations account for a very small number of ALS cases (~2.5%) so the amount human tissue available is very limited. However, it is clear that a combination of these studies would greatly enhance our understanding of SOD1 metallation with respect to ALS.

## Conclusions

Both metal ion homeostasis and aggregate formation have been implicated in the disease progression of ALS. This study used a cell culture model of SOD1-ALS in order to examine metal ion homeostasis and aggregate formation in a systematic manner within the cellular environment. Results showed an increase in copper content when WT SOD1 was overexpressed, which decreased in cells transfected with mutant SOD1 showing a disruption in copper homeostasis. Additionally, the aggregates were found to be metal-deficient, supporting the hypothesis that they form without metal-binding that stabilizes the protein. Understanding the role that metals play in SOD1 aggregate can lead to the development of improved drug treatments to prevent aggregate formation and hopefully prevent the progression of the disease.

## Conflict of interest statement

The authors declare that the research was conducted in the absence of any commercial or financial relationships that could be construed as a potential conflict of interest.
